# Topological kink plasmons on magnetic-domain boundaries

**DOI:** 10.1038/s41467-019-12092-x

**Published:** 2019-10-08

**Authors:** Dafei Jin, Yang Xia, Thomas Christensen, Matthew Freeman, Siqi Wang, King Yan Fong, Geoffrey C. Gardner, Saeed Fallahi, Qing Hu, Yuan Wang, Lloyd Engel, Zhi-Li Xiao, Michael J. Manfra, Nicholas X. Fang, Xiang Zhang

**Affiliations:** 1 Nanoscale Science and Engineering Center, University of California, Berkeley, CA 94706 USA; 2 Center for Nanoscale Materials, Argonne National Laboratory, Argonne, IL 60439 USA; 3 Department of Physics, Massachusetts Institute of Technology, Cambridge, MA 02139 USA; 4 National High Magnetic Field Laboratory, Tallahassee, FL 32310 USA; 5 Microsoft Quantum Purdue and Birck Nanotechnology Center, Purdue University, West Lafayette, IN 47907 USA; 6 Department of Physics and Astronomy and Birck Nanotechnology Center, Purdue University, West Lafayette, IN 47907 USA; 7 Department of Mechanical Engineering, Massachusetts Institute of Technology, Cambridge, MA 02139 USA; 8 Material Science Division, Argonne National Laboratory, Argonne, IL 60439 USA; 9 Microsoft Quantum Purdue, Department of Physics and Astronomy, Birck Nanotechnology Center, Schools of Electrical and Computer Engineering and Materials Engineering, Purdue University, West Lafayette, IN 47907 USA; 10 Faculties of Sciences and Engineering University of Hong Kong, Hong Kong SAR, PR China

**Keywords:** Nanophotonics and plasmonics, Two-dimensional materials, Electronic and spintronic devices

## Abstract

Two-dimensional topological materials bearing time reversal-breaking magnetic fields support protected one-way edge modes. Normally, these edge modes adhere to physical edges where material properties change abruptly. However, even in homogeneous materials, topology still permits a unique form of edge modes – kink modes – residing at the domain boundaries of magnetic fields within the materials. This scenario, despite being predicted in theory, has rarely been demonstrated experimentally. Here, we report our observation of topologically-protected high-frequency kink modes – kink magnetoplasmons (KMPs) – in a GaAs/AlGaAs two-dimensional electron gas (2DEG) system. These KMPs arise at a domain boundary projected from an externally-patterned magnetic field onto a uniform 2DEG. They propagate unidirectionally along the boundary, protected by a difference of gap Chern numbers ($\pm1$) in the two domains. They exhibit large tunability under an applied magnetic field or gate voltage, and clear signatures of nonreciprocity even under weak-coupling to evanescent photons.

## Abstract (web summary)

Topological kink modes are peculiar edge excitations that take place at domain boundaries of magnetic fields inside homogeneous materials. Here, the authors experimentally observe kink magnetoplasmons in a 2D electron gas using custom-shaped strong permanent magnets on top of a GaAs/AlGaAs heterojunction.

## Introduction

Topologically protected one-way edge modes can exist in two-dimensional systems under a time-reversal-breaking magnetic field^1–3^. Such modes usually arise at physical edges where material properties undergo a sudden change. Nevertheless, another type of topological edge modes, termed topological kink modes, can exist at magnetic-domain boundaries inside an otherwise homogeneous system^1–8^. Intuitively, this system can be viewed as composed of two effective materials, distinguished by the sign of magnetic field, and separated by a synthetic edge from the domain boundary. While time-reversal-preserving kink modes have been observed in both the low-frequency fermionic (electronic) and high-frequency bosonic (photonic) valley-Hall systems^9,10^, time-reversal-broken kink modes have only been observed in fermionic systems^11^. Various bosonic (photonic, plasmonic, magnonic, and excitonic^12–19^) counterparts have yet to be demonstrated experimentally.

A two-dimensional electron gas (2DEG) in a high-mobility GaAs/AlGaAs heterojunction^20,21^ is an ideal platform to realize kink modes. Under a perpendicular magnetic field, the 2DEG hosts magnetoplasmons (MPs)—electron-density oscillations sustained by the longitudinal Coulomb force and subjected to a transverse Lorentz force—covering a broad spectral range from radio to microwave frequencies. These MPs embody a prototypical band topology of bosonic excitations^5,17^. The magnetic field opens a topological gap for the bulk MPs up to a cyclotron frequency^22,23^. Topologically protected edge magnetoplasmons (EMPs) bridge the bulk gap and propagate unidirectionally along system’s boundaries^24–27^.

Previous studies of EMPs in any 2DEG systems all relied on sharp termination of electron density $n({\bf{r}})$ at sample edges^28–30^. In this work, we achieve an innovative device design that ensures a constant electron density ${n}_{0}$ throughout the main area of 2DEG, but contains a space-varying magnetic field ${\bf{B}}({\bf{r}})=B({\bf{r}})\hat{{\bf{e}}}_z$^31–33^ in the 2DEG. This magnetic field is produced by a custom-shaped NdFeB strong permanent magnet placed atop the GaAs/AlGaAs heterojunction. It generates a magnetic field of opposite signs about $\pm 0.15$ T in the two domains, sufficient to produce a sizable MP bulk gap in each domain. The high electron mobility $\mu \sim 1{0}^{7}\ {{\rm{cm}}}^{2}\ {{\rm{V}}}^{-1}\ {{\rm{s}}}^{-1}$ of our 2DEG affords an ultra-long relaxation time up to hundreds of picoseconds and ultra-low damping rate down to a few gigahertz, superior to most existing 2DEG systems^24,34,35^. We experimentally demonstrate the existence of KMPs and their nonreciprocal nature, by measuring the resonance absorption spectrum in the 1–10 GHz frequency range. We find that the excitation frequencies of KMPs exhibit a unique dependence on an additionally applied magnetic field or gate voltage, differing substantially from the conventional EMPs. Our theoretical calculation and experimental observation show good mutual agreement.

## Results

### Device

**Fig. 1:**
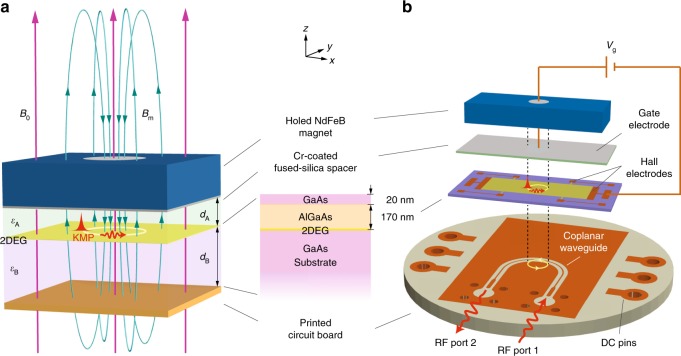
Topological magnetoplasmonic device. **a** Conceptual layout. **b** Device design and PCB layout. The 2DEG is formed at the interface of a GaAs/AlGaAs heterojunction, cladded between a fused-silica spacer and the substrate, and finally enclosed in a metallic cavity. A holed NdFeB magnet on the top provides an oppositely signed magnetic field at the 2DEG to permit kink magnetoplasmons (KMPs) traveling unidirectionally along the magnetic-domain boundary. Microwaves transmitted along the coplanar waveguide on a printed circuit board excite the KMPs. An applied uniform magnetic field ${B}_{0}$ or a gate voltage ${V}_{\text{g}}$ can tune the KMPs

Figure 1 illustrates the layout of our topological magnetoplasmonic device. Conceptually (Fig. 1a), a 2DEG in a GaAs/AlGaAs heterojunction (see the “Methods” section) is cladded above and below by a fused-silica spacer and a GaAs substrate, respectively, of thicknesses $d_{\mathrm{A}}=100$μm and $d_{\mathrm{B}}=150$μm, and permittivities $\varepsilon_{\mathrm{A}}=3.8$ and $\varepsilon_{\mathrm{B}}=12.8$. This dielectric–2DEG–dielectric structure is enclosed in a metallic cavity along $z$, terminated at the spacer’s top and substrate’s bottom. A holed NdFeB permanent magnet installed atop the cavity projects a circular magnetic field ${{\bf{B}}}_{\text{m}}({\bf{r}})={B}_{\text{m}}(r)\hat{{\bf{e}}}_{z}$ onto the 2DEG. The sign of ${B}_{\text{m}}(r)$ changes sharply across the projection of the hole’s radius, $a=0.75$ mm, producing the adjacent oppositely signed magnetic domains (see the “Methods” section). The entire 2DEG is additionally exposed to a tunable uniform magnetic field ${{\bf{B}}}_{0}={B}_{0}\hat{{\bf{e}}}_{z}$ from a superconducting coil, allowing an overall shift of the field profile.

In practice (Fig. 1b), the heterojunction sample has a $12\times 6\ {{\rm{mm}}}^{2}$ rectangular footprint. A $9\times 3\ {{\rm{mm}}}^{2}$ Hall bar is fabricated on it, allowing in situ measurements and control of the 2DEG electron concentration ${n}_{0}$. The fused silica spacer is topped by a 100 nm-thick e-beam evaporated Cr-coating, serving simultaneously as upper cavity wall and gate electrode^36,37^. A gate voltage of ${V}_{\text{g}} \sim \pm100$ V can be applied across the Cr-coating–Hall bar junction to tune the electron concentration. The sample–spacer–magnet assembly is glued by poly(methyl methacrylate) (PMMA) onto a customized Cu printed circuit board (PCB) with a 5 μm Ni and 200 nm Au surface finish. The PCB hosts a coplanar waveguide (CPW) connecting RF Ports 1 and 2 with mini-SMP connectors^36^. By design, the CPW has a 50 $\Omega$ impedance with the sample–magnet assembly loaded. The CPW signal line is aligned tangentially to the projected circle from the hole of magnet so as to maximize the microwave–KMP coupling.

### Theory

The essential physics of MPs can be captured by the continuity equation and a constitutive equation including the longitudinal Coulomb and transverse Lorentz forces:

1 $$\omega \rho ({\bf{r}},\omega )=-{\rm{i}}\nabla \cdot {\bf{j}}({\bf{r}},\omega ),$$

2 $$\omega {\bf{j}}({\bf{r}},\omega )=-{\rm{i}}\frac{{e}^{2}}{{m}_{* }}n({\bf{r}})\nabla \Phi ({\bf{r}},\omega )-{\rm{i}}\omega_ {\mathrm{{c}}}({\bf{r}}){\bf{j}}({\bf{r}},\omega )\times \hat{{\bf{e}}}_z.$$

Here, ${\bf{j}}$ and $\rho$ are the surface current and charge densities, evaluated at frequencies $\omega$ and in-plane positions ${\bf{r}}$. $\Phi ({\bf{r}},\omega )=\int V({\bf{r}}-{\bf{r}}' )\rho ({\bf{r}}',\omega )\ {{\rm{d}}}^{2}{{\bf{r}}'}$ is the self-consistent potential due to the (screened) Coulomb interaction $V$. $\omega_{\mathrm{{{c}}}}({\bf{r}})=eB({\bf{r}})/{m}_{* }c$ is a space-varying cyclotron frequency, with ${m}_{* }=0.067{m}_{e}$ the electron effective mass. As elaborated below, even with a constant electron density $n({\bf{r}})={n}_{0}$, topologically protected KMPs can reside at sign-changing magnetic domain boundaries defined solely by the spatial profile $B({\bf{r}})$ and $\omega_{\mathrm{{{c}}}}({\bf{r}})$^5^.

The total magnetic field, $B(r)={B}_{0}+{B}_{\text{m}}(r)$, is the sum of a tunable, uniform field ${B}_{0}$ from the superconducting coil, and a fixed, $r$-dependent field ${B}_{\text{m}}(r)$ from the holed NdFeB permanent magnet. The latter is well-approximated by

3 $${B}_{\mathrm{m}}(r)\simeq \bar{B}_{\mathrm{m}}+{\rm{sgn}}(r-a)\Delta B_{\mathrm{m}}.$$

Here, $\Delta B_{\mathrm{m}}$ contributes an equal-magnitude sign-changing jump at $r=a\approx 0.75\ {\rm{mm}}$, while $\bar{B}_{\mathrm{m}}$ accounts for a small, overall shift due to the small distance between magnet and 2DEG. By a combination of finite-element simulations and room-temperature Hall-probe measurements on the surface of magnet, we infer the low-temperature values of each as $\Delta B_{\mathrm{m}}\approx 0.14$ T and $\bar{B}_{\mathrm{m}}\approx 0.01$ T (see the “Methods” section).

The in-plane Coulomb interaction, which determines the plasmonic frequency scale, is screened by the dielectric response of the materials cladding the 2DEG above ($\varepsilon_{\mathrm{{A}}}$) and below ($\varepsilon_{\mathrm{{B}}}$); in momentum space, it takes the form^26,27^

4 $$V(q)=\frac{2\pi }{q}\beta (q)=\frac{2\pi }{q}\frac{2}{\varepsilon_ {\mathrm{A}}\coth (qd_{\mathrm{A}})+\varepsilon_{\mathrm{B}}\coth (qd_{\mathrm{B}})},$$

with $\beta (q)$ being the $q$-dependent screening function. The scalar potential and surface charge density are related by $\Phi (q)=V(q)\rho (q)$. The eigenmodes of the system consistent with Eqs. (1) and (2) are eigenstates of a $3\ \times \ 3$ Hamiltonian ${\mathcal{H}}$ with operator elements^5,17^. In the circularly symmetric “potential” of Eq. (3), the eigenmodes decompose according to ${{\mathcal{R}}}_{m}(r){{\rm{e}}}^{{\rm{i}}m\varphi }$ with azimuthal angle $\varphi$ and angular wavenumber $m\in {\mathbb{Z}}$. The radial function ${{\mathcal{R}}}_{m}(r)$ can be expanded by the Bessel functions with radial wavenumbers ${q}_{mn}$, $n\in {{\mathbb{Z}}}^{+}$, which enter the Coulomb interaction Eq. (4) (see the “Methods” section).

**Fig. 2:**
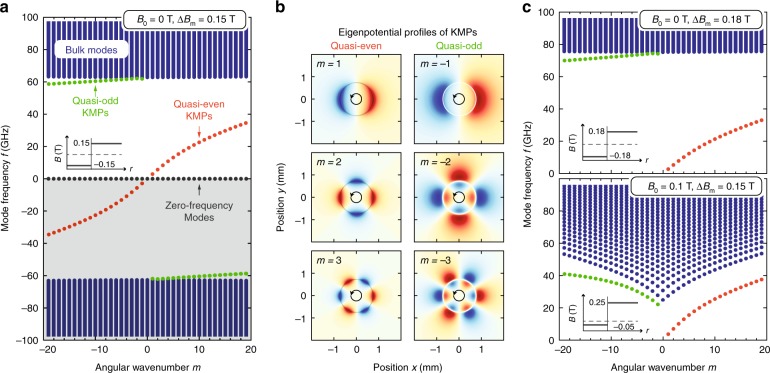
Theoretical calculation of topological kink magnetoplasmons (KMPs). **a** Magnetoplasmonic dispersion of bulk and edge modes with angular wavenumber $m$ (external field ${B}_{0}=0$ T, material-induced field $\Delta B_{\mathrm{m}}=0.15$ T). **b** Eigenpotential profiles of quasi-even and quasi-odd KMPs for $|m|=\text{1, 2, and 3}$ (${B}_{0}$ and $\Delta B_{\mathrm{m}}$ as in **a**). **c** Magnetoplasmonic dispersion for ${B}_{0}=0$ T, $\Delta B_{\mathrm{m}}=0.18$ T and ${B}_{0}=0.1$ T, $\Delta B_{\mathrm{m}}=0.15$ T. Insets indicate the total magnetic field profile $B(r)$ (vertical gray line, $r=a$). ${n}_{0}=1\times 1{0}^{11}\ {{\rm{cm}}}^{-2}$ and $\bar{B}_{\mathrm{m}}=0$ T in all panels

Figure 2a illustrates the magnetoplasmonic dispersion of bulk MP and KMP modes for ${n}_{0}=1\times 1{0}^{11}\ {{\rm{cm}}}^{-2}$, ${B}_{0}=\bar{B}_{\mathrm{m}}=0$ T, and $\Delta B_{\mathrm{m}}=0.15$ T. The spectrum exhibits particle–hole symmetry, i.e. ${\omega }_{nm}=-{\omega }_{n,-m}$, with a zero-frequency band describing static modes^5^. The bulk MPs in each magnetic domain contains a gap from zero frequency to the cyclotron frequency $|\omega_{{\mathrm{{c}}}}(r)|=e|B(r)|/{m}_{* }c$, in this case about 60 GHz. The band topology of each domain, considered as an extended bulk, is characterized by a topological invariant, the Chern number, equaling $C=-{\rm{sgn}}B(r)={\rm{sgn}}(a-r)=\pm 1$^5^. The associated gap Chern number $\bar{C}$ also equals $\pm 1$; its difference across domains of oppositely directed magnetic fields is $\Delta \bar{C}=2$, dictating the existence of two unidirectional edge states localized at $r=a$. This is a manifestation of a bulk-edge correspondence: the two domains of the 2DEG are topologically distinct (but share a common bulk band gap) due to their opposite magnetic biases; the emergence of edge-localized kink magnetoplasmons furnishes a continuous transition between the two domains by crossing and thereby connecting the bulk gap, allowing a change of topology from one domain to the other.

These conclusions are manifest in Fig. 2a, b from the existence of quasi-even and quasi-odd KMP branches (so named due to their asymptotic association with the even and odd KMPs of a linear domain boundary). Both are unidirectional and exhibit increasing localization with incrementing angular wavenumbers $m$. They differ from the conventional EMPs even at the microscopic level. For EMPs, the electron-density waves hit a physical barrier where the momentum is immediately reversed. For KMPs, however, the electron-density waves hit a magnetic barrier where the Lorentz force is reversed. There are classical analogies to the two kinds of KMPs; they are topologically equivalent to the equatorial Kelvin and Yanai waves of the ocean and atmosphere with a Coriolis parameter replacing $B(r)$^38^.

Figure 2c investigates the dispersion for increased $\Delta B_{\mathrm{m}}$ (from 0.15 to 0.18 T) and ${B}_{0}$ (from 0 to 0.1 T). Comparing to Fig. 2a, increasing $\Delta B_{\mathrm{m}}$ widens the bandgap and decreases the frequencies of the quasi-even KMPs. Conversely, increasing ${B}_{0}$ (but maintaining *B*_0_ < Δ*B*_m_) reduces the overall gap—since the cyclotron frequency is lowered in the inner domain—and increases the excitation frequencies of the quasi-even KMP. This latter behavior further distinguishes our new KMPs from the traditional EMPs which shift in the opposite direction with increasing ${B}_{0}$^24,26^. The quasi-odd KMP branch in Fig. 2c appears as though they are not gapless. This puzzle, however, is remedied at larger $|m|$ where the quasi-odd dispersion curve turns downwards towards the zero-frequency modes (see the “Methods” section), same as Fig. 2a, c. This reinstates an asymptotically gapless behavior fulfilling the topological requirements of MP systems^5^.

### Experiment

We next seek experimental evidence for the theoretically predicted KMPs specific to our device. The device is inserted into a He-3 cryostat running at 0.5 K. An Agilent E5071C network analyzer (NA) is used to acquire power transmission ${S}_{21}$ (Port 1 to 2) and ${S}_{12}$ (Port 2 to 1) in the frequency range 300 kHz to 20 GHz^18,36,37^. Our focused frequency range is limited to 1–10 GHz, beyond which the cables and NA suffer high loss and noise, prohibiting acquisition of clear signals. Referring to Fig. 2a, c, we expect to observe characteristic absorption associated with the $m=1$ and $2$ quasi-even KMPs. Note that the quasi-odd KMPs fall into our spectral range only at extremely large (angular) momenta. Their non-dispersive nature will only produce a broad absorption background in the spectrum with no resolvable resonances.

**Fig. 3:**
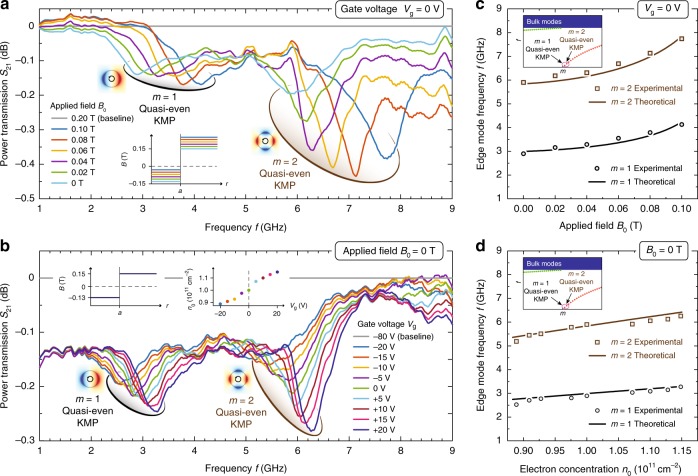
Experimental observation of topological kink magnetoplasmons (KMPs). **a** and **b** Measured power transmission ${S}_{21}$ (normalized to indicated baselines) as a function of frequency for **a**, varying applied uniform fields ${B}_{0}$ ($V_{\mathrm{{g}}}=\text{0 V}$) and **b**, varying gate voltages $V_{\mathrm{g}}$ (${B}_{0}=\text{0 T}$). The characteristic absorption dips correspond to quasi-even KMPs with angular wavenumber $m=\text{1}$ and 2, as indicated. Insets depict the (idealized) total magnetic field profile $B(r)$ across the domains, as well as the measured dependence of the electron concentration ${n}_{0}$ with $V_{\mathrm{g}}$ (from independent Hall transport measurements). **c** and **d** Comparison between experimental observations and theoretical calculations. Dependence of KMP frequencies with **c**, applied field ${B}_{0}$ ($V_{\mathrm{g}}=\text{0 V}$, ${n}_{0}=\text{1}\times {\text{10}}^{\text{11}}\ {{\rm{cm}}}^{-\text{2}}$) and **d** electron concentration ${n}_{0}$ (${B}_{0}=\text{0 T}$)

In the first series of measurements, we keep the gate grounded, $V_{\mathrm{g}}=0$ V, and investigate the influence of the applied magnetic field ${B}_{0}$ on the resonant absorption of quasi-even KMPs in ${S}_{21}$ (Fig. 3a). All signals are divided by a reference (denoted baseline) and processed with five-point curve smoothing (refer to Supplementary Fig. 1). Here, we choose ${B}_{0}=0.2$ T as baseline, which provides a high suppression of unwanted low-frequency bulk modes, without exerting too great a torque on the magnet–sample assembly. For every ${S}_{21}$-spectrum in Fig. 3a, each reflecting a single applied field in the range ${B}_{0}=0$–$0.1$ T, we observe two well-defined absorptive resonances, corresponding to the $m=1\,\text{and}\,2$ right-circulating quasi-even KMPs. Spanning frequencies from 3 to 4 GHz and 6 to 8 GHz, they exhibit linewidths of 1 to 2 GHz, roughly consistent with the Hall-probe inferred DC damping rate $\gamma \approx 2.6$ GHz. Figure 3c compares the measured and theoretically predicted resonance frequencies. We make two observations: first, measurements and theory agree well, in the absence of fitting parameters; second, the excitation frequencies increase monotonously with increasing ${B}_{0}$, which unambiguously differentiates our magnetically defined KMPs from conventional EMPs.

In the second series of measurements, we fix the applied magnetic field ${B}_{0}=0$ T, and explore the KMPs’ dependence on the gate voltage $V_{\mathrm{g}}$ (Fig. 3b). The baseline is chosen at $V_{\mathrm{g}}=-80$ V, which corresponds to an essentially electron-depleted 2DEG supporting no plasmonic modes. Once more, every spectrum in Fig. 3b, each now corresponding to distinct gate voltages in the range $V_{\mathrm{g}}=-20$ to $+20$ V, exhibits two clear absorptive resonances associated with the $m=1\,\text{and}\,2$ quasi-even KMPs. Increasing the gate voltage (or, equivalently, the electron concentration ${n}_{0}$) increases the KMP frequency, as expected. Moreover, the extinction depth of each resonance also increases with the $V_{\mathrm{g}}$. This is consistent with the $f$-sum rule^39^ which dictates a linear increase of integrated extinction with increased ${n}_{0}$ (disregarding the negligible spectral dispersion in the microwave–KMP coupling). Comparing theoretical and experimental observations (Fig. 3d) we again find good agreement.

**Fig. 4:**
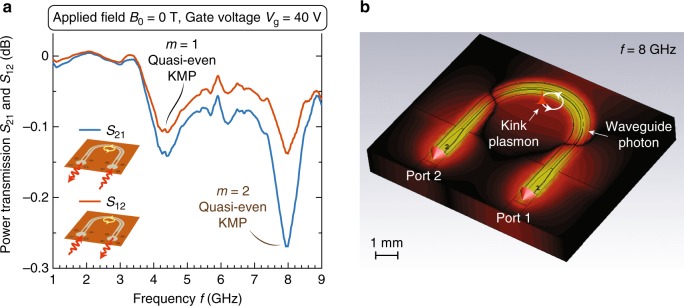
Nonreciprocal transmission of kink magnetoplasmons (KMPs). **a** Measured power transmission along ${S}_{21}$ (“easy-coupling”) and ${S}_{12}$ (“hard-coupling”) directions, respectively, with gate voltage $V_{\mathrm{g}}=40$ V and zero applied magnetic field. The distinct absorption depths manifests the nonreciprocal nature of the $m=\text{1}$ and 2 quasi-even KMPs. **b** Full-wave simulated mode profile of long-wavelength microwave photons in the coplanar waveguide, in comparison to the small circle along which short-wavelength one-way KMPs propagate

Using the same sample and magnet, we examine the nonreciprocal properties of the KMPs in Fig. 4a, to explicitly demonstrate the underlying unidirectional character of the KMPs. Since the KMPs are right-circulating in the bandgap (Fig. 2), ${S}_{21}$ and ${S}_{12}$ correspond to the “easy-coupling” and “hard-coupling" directions, respectively, of our device (Fig. 1b). Each coupling direction is normalized separately, with baselines taken at ${B}_{0}=0.2$ T. The 2DEG is gated by $V_{\mathrm{g}}=40$ V, ensuring a pronounced extinction depth, and the applied magnetic field is turned off ${B}_{0}=0$ T. In this configuration, the $m=1$ and $2$ quasi-even KMPs exist at 4.2 and 8.0 GHz, respectively. Comparing ${S}_{21}$ and ${S}_{12}$ we observe distinct asymmetry of extinction depth at each resonance, with ${S}_{12}$ exhibiting shallower extinction. This asymmetry is indicative of the unidirectional character of the KMPs.

## Discussion

The isolation ratio ${S}_{21}/{S}_{12}={({S}_{21}-{S}_{12})|}_{\text{dB}}$ attained from our current experiment is limited by the evanescent-photon–plasmon coupling technique. It is important to stress that the KMPs themselves are always unidirectional, independent of the properties of the photons used to probe them. In order to observe a sharp isolation contrast, the probe’s extent should in principle be much smaller than the length scale of the edge (or kink) modes^12,28,30,40^. However, the probing technique compatible with our device here employs the evanescent field from CPW photons, which are more delocalized than the KMPs.

To be quantitative, we have performed a full-wave simulation for the wavelengths and mode profiles of microwave photons along the CPW, as shown in Fig. 4b. At $f=8$ GHz excitation frequency (corresponding to the $m=2$ quasi-even mode resonance), the waveguide photon wavelength is about 1 cm, whereas the KMP (angular) wavelength is only about 2 mm. As a result, the KMPs only see a slowly varying nearly quasistatic photon field with $< \pi / 5$ phase variation across their circulating diameter. This significantly limits the attainable isolation ratio, since the waveguide photons, regardless of direction, couples weakly with both KMPs. Nonetheless, despite this weak coupling between the photon probe and KMPs, the nonreciprocal nature of KMPs is evident.

In summary, we have experimentally realized high-frequency topological kink modes, kink magnetoplasmons (KMPs), in a unique magnetoplasmonic device featuring oppositely biased magnetic domains. The KMPs localize at the magnetic domain boundaries in the otherwise homogeneous two-dimensional electron gas (2DEG). Our experimental observation show good agreement with theoretical calculation. The demonstrated KMP architecture can be generalized to accommodate more complex magnetic patterns, and could be useful for novel integrated topological circuits ^13,18,19^.

## Methods

### 2DEG sample growth and characterization

**Fig. 5:**
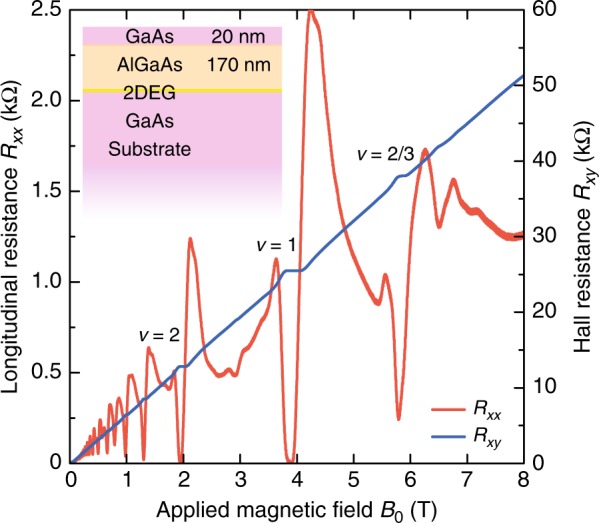
Quantum Hall measurement of the GaAs/AlGaAs 2DEG sample at 0.3 K and zero gate voltage. The inset shows the out-of-plane structure of the sample

Our sample is a single-interface GaAs/${{\rm{Al}}}_{x}$Ga${}_{1-x}$As ($x=0.22$) heterojunction grown by molecular beam epitaxy (MBE) on a 500 $\mu \ {\rm{m}}$-thick GaAs wafer. After the growth, the sample is back-polished down to 100 $\mu \ {\rm{m}}$ thick in order to enhance the evanescent microwave coupling. The MBE growth consists of a 500 nm-thick GaAs layer followed by a 170 nm-thick ${{\rm{Al}}}_{x}{{\rm{Ga}}}_{1-x}$As ($x=0.22$) spacer and a 20 nm GaAs cap layer to prevent oxidization of the AlGaAs barrier. It is delta-doped with Si doping concentration $1.6\times 1{0}^{12}\ {{\rm{cm}}}^{-2}$ at a setback of 120 nm above the GaAs/AlGaAs interface containing 2DEG. The 2DEG lies 190 nm below top surface. The electron concentration ${n}_{0}=0.95\times 1{0}^{11}\ {{\rm{cm}}}^{-2}$ and mobility $\mu =8.6\times 1{0}^{6}\ {{\rm{cm}}}^{2}\ {{\rm{V}}}^{-1}\ {{\rm{s}}}^{-1}$ are extracted from our Hall measurement at $T=0.3$ K in dark. In our actual microwave experiment at 0.5 K, the typical zero-gate electron concentration is measured to be about $1\times 1{0}^{11}\ {{\rm{cm}}}^{-2}$. This number is used in our calculation. The uniform magnetic field ${B}_{0}$ is supplied by a superconductor coil. In the absence of the holed NdFeB magnet, it can safely reach above 7 T, enabling a quantum-Hall measurement to characterize the sample (see Fig. 5). When the NdFeB magnet is present, the applied field is limited by practical concerns to at most 0.5 T, beyond which a huge magnetic torque is exerted onto the magnet, risking damage to the sample.

### NdFeB magnet design and characterization

**Fig. 6:**
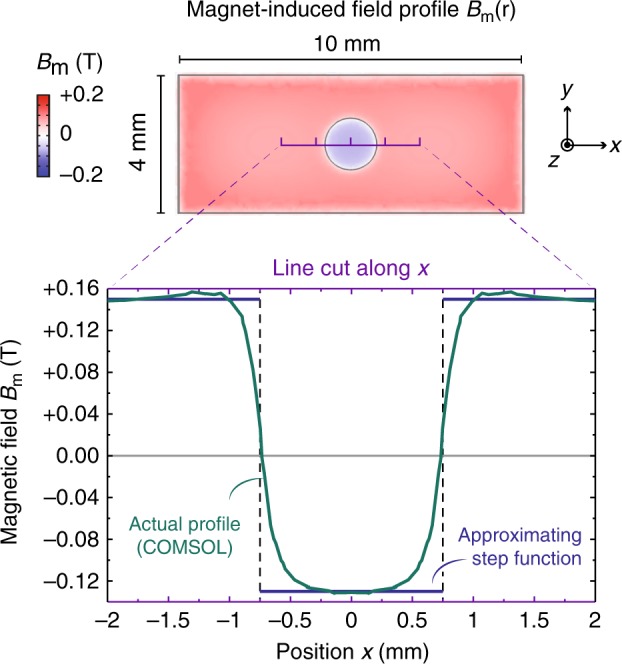
Calculated magnetic field profile of the holed NdFeB magnet at low temperature. The simulation parameters are obtained from the Hall-probe measurement at room temperature and extrapolated into low temperature following the literature^41–43^

The NdFeB magnet is 1 mm long, 4 mm wide, and 1 mm thick, and the hole radius is 0.75 mm. It is produced by sintering NdFeB powders in a custom mold and subsequently magnetizing it along the thickness direction. At room temperature, Hall-probe measurements indicate that the holed magnet provides ~$\pm 0.18$ T remanent magnetic field in the surface area inside and outside the hole. With this value, and taking into account the known anisotropic reduction of the magnetism of NdFeB at cryogenic temperatures ^41–43^, we are able to simulate out the magnetic field profile over the entire magnet at low temperature (see Fig. 6) using a finite-element software (Comsol Multiphysics). From the results, we infer that the two key parameters of Eq. (2), namely, a sign-changing field strength $\Delta B_{\mathrm{{{m}}}}\approx \pm 0.14$ T and a overall shift $\bar{B}_{\mathrm{{{m}}}}\approx 0.01$ T. These are the values used in our theoretical calculations in Fig. 2c, d, demonstrating good agreement between theory and experiment with no fitting parameters.

### Theoretical development and computational scheme

The evanescent nature of KMPs and the presence of encapsulating metals, which screen away the long-range part of Coulomb interaction, allow us to focus on the region around and inside the circle $r\, \lesssim \,a=0.75\ {\rm{mm}}$. We can legitimately take a circularly symmetric model system cut off at a radius $R=10\ {\rm{mm}}\,\gg a$, where the scalar potential $\Phi$ is grounded $\Phi (r=R,\varphi )=0$. This truncation does not affect the evanescent KMPs that localize around $r=a$, far away from the truncation region.

The associated eigenproblem can be conveniently expressed in a chiral representation^5^

5 $$\omega j_{\mathrm{R}}(r,\varphi )=+\omega_{\mathrm{{{c}}}}(r)j_{\mathrm{R}}(r,\varphi )+\frac{{e}^{2}{n}_{0}}{{\omega }_{0}{m}_{* }}\frac{{{\rm{e}}}^{-{\rm{i}}\varphi }}{{\rm{i}}\sqrt{2}}{\partial }_{-}j_{\mathrm{D}}(r,\varphi ),$$

6 $$\omega j_{\mathrm{D}}(r,\varphi )={\omega }_{0}\hat{V}\frac{{{\rm{e}}}^{+{\rm{i}}\varphi }}{{\rm{i}}\sqrt{2}}{\partial }_{+}j_{\mathrm{R}}(r,\varphi )+{\omega }_{0}\hat{V}\frac{{{\rm{e}}}^{-{\rm{i}}\varphi }}{{\rm{i}}\sqrt{2}}{\partial }_{-}j_{\mathrm{L}}(r,\varphi ),$$

7 $$\omega j_{\mathrm{L}}(r,\varphi )=-\omega_{\mathrm{c}}(r)j_{\mathrm{L}}(r,\varphi )+\frac{{e}^{2}{n}_{0}}{{\omega }_{0}{m}_{* }}\frac{{{\rm{e}}}^{+{\rm{i}}\varphi }}{{\rm{i}}\sqrt{2}}{\partial }_{+}j_{\mathrm{D}}(r,\varphi ),$$

in which ${\partial }_{\pm }\equiv {\partial }_{r}\pm \frac{{\rm{i}}}{r}{\partial }_{\varphi }$. The basic field components are the right-circulating current $j_{\mathrm{R}}\equiv \frac{1}{\sqrt{2}}({j}_{r}-{\rm{i}}{j}_{\varphi }){{\rm{e}}}^{-{\rm{i}}\varphi }$, the left-circulating current $j_{\mathrm{L}}\equiv \frac{1}{\sqrt{2}}({j}_{r}+{\rm{i}}{j}_{\varphi }){{\rm{e}}}^{+{\rm{i}}\varphi }$, and the “scalar-potential (density-fluctuation)" current $j_{\mathrm{D}}\equiv {\omega }_{0}\Phi$. Here, ${\omega }_{0}\equiv \sqrt{{e}^{2}{n}_{0}/{m}_{* }R}$ is a characteristic plasmon frequency, $\omega_{\mathrm{{c}}}(r)=eB(r)/{m}_{* }c$ is the $r$-dependent cyclotron frequency, and $\hat{V}$ is the Coulomb interaction operator,

8 $$\hat{V}\rho (r,\varphi )={\int }_{\! 0}^{R}r' {\rm{d}}r' {\int }_{\! 0}^{2\pi }{\rm{d}}{\varphi}' V(|{\boldsymbol{r}}-{{\boldsymbol{r}}'} |)\rho (r',{\varphi}' ),$$

with $V(|{\boldsymbol{r}}-{{\boldsymbol{r}}'} |)$ being the screened in-plane Coulomb interaction in real space and relating to Eq. (4) by a Fourier transform.

The eigensolutions with a given angular wavenumber $m$ and obeying the hard-wall boundary condition are linear expansion of Bessel functions

9 $${j}_{s}(r,\varphi )=\left[\mathop{\sum}\limits_{n=1}^{N\to \infty }{A}_{n,s}{{\rm{J}}}_{m+s}({q}_{mn}r)\right]{{\rm{e}}}^{+{\rm{i}}(m+s)\varphi }.$$

Here $s=-1,0,+1$ resembles a spin index referring to the $j_{\mathrm{R}}$, $j_{\mathrm{D}}$, $j_{\mathrm{L}}$ components, respectively. ${q}_{mn}={\zeta }_{mn}/R$ are discretized radial wavenumbers with ${\zeta }_{mn}$ denoting the $n$th zero of the $m$th order Bessel function ${{\rm{J}}}_{m}(\zeta )$. In practice, the expansion is truncated at a finite $N$, determined by the desired spectral resolution ($N=2000$ in our calculations). In this discrete cylindrical-wave basis, the screened Coulomb interaction relates $\rho$ and $\Phi$ by $\Phi ({q}_{mn})=V({q}_{mn})\rho ({q}_{mn})$, with $V(q)$ defined by Eq. (4).

The matrix-form eigen-equation in the cylindrical-wave bases reads

10 $$\frac{\omega }{{\omega }_{0}}\left(\begin{array}{c}{{\mathcal{A}}}_{+1}\\ {{\mathcal{A}}}_{0}\\ {{\mathcal{A}}}_{-1}\end{array}\right)=\left(\begin{array}{ccc}+{\mathcal{W}}&+\frac{{q}_{mn}R}{{\rm{i}}\sqrt{2}}{\mathcal{I}}&0\\ -\frac{2\pi \beta ({q}_{mn})}{{\rm{i}}\sqrt{2}}{\mathcal{I}}&0&+\frac{2\pi \beta ({q}_{mn})}{{\rm{i}}\sqrt{2}}{\mathcal{I}}\\ 0&-\frac{{q}_{mn}R}{{\rm{i}}\sqrt{2}}{\mathcal{I}}&-{\mathcal{W}}\end{array}\right)\left(\begin{array}{c}{{\mathcal{A}}}_{+1}\\ {{\mathcal{A}}}_{0}\\ {{\mathcal{A}}}_{-1}\end{array}\right).$$

Here ${{\mathcal{A}}}_{s}={({A}_{1,s},{A}_{2,s},\ldots,{A}_{N,s})}^{\text{T}}$, ${\mathcal{I}}$ is an $N\times N$ identity matrix, ${\mathcal{W}}$ is an $N\times N$ full matrix determined by the magnetic-field profile. If $B(r)={B}_{0}$, then ${\mathcal{W}}=(\omega_{\mathrm{c}}/{\omega }_{0}){\mathcal{I}}$ is diagonal with the constant cyclotron frequency $\omega_{\mathrm{c}}=e{B}_{0}/{m}_{* }c$, and the usual bulk MP modes can be recovered^5^.

The radially varying magnetic field in our problem results in scattering between different radial indices $n$ (within the $s=-1$ and $+1$ chiral subspace), which localizes the edge modes to the magnetic-domain boundary. The matrix ${\mathcal{W}}$ can be determined from the expansion

11 $$\omega_{\mathrm{c}}(r){{\rm{J}}}_{m-1}({q}_{mn}r)\equiv {\omega }_{0}\mathop{\sum}\limits_{n{\prime} }{W}_{nn{\prime} }{{\rm{J}}}_{m-1}({q}_{mn{\prime} }r),$$

12 $$\omega_{\mathrm{c}}(r){{\rm{J}}}_{m+1}({q}_{mn}r)\equiv {\omega }_{0}\mathop{\sum }\limits_{n{\prime} }{W}_{nn{\prime} }{{\rm{J}}}_{m+1}({q}_{mn{\prime} }r),$$

with ${W}_{nn{\prime}}$ denoting the elements of ${\mathcal{W}}$. After lengthy manipulations involving Bessel integrals^44^, we obtain

13 $${\mathcal{W}}=\frac{e}{{\omega }_{0}{m}_{*}c}\left[2{\Delta} B_{{\mathrm{m}}}{{\mathcal{Y}}}^{-1}{\mathcal{X}}+({B}_{0}+{\bar{B}}_{\rm{m}}-{\Delta} B_{\rm{m}}){\mathcal{I}}\right],$$

where ${\mathcal{X}}$ and ${\mathcal{Y}}$ are $N\times N$ matrices with elements

14 $${X}_{nn{\prime} } 	={\int }_{0}^{\tilde{a}}\tilde{r}{\rm{d}}\tilde{r}{{\rm{J}}}_{m-1}({\zeta }_{mn}\tilde{r}){{\rm{J}}}_{m-1}({\zeta }_{mn{\prime} }\tilde{r})\\ 	=\left\{\begin{array}{l}\frac{\tilde{a}}{{\zeta }_{mn}^{2}-{\zeta }_{mn{\prime} }^{2}}\left\{\begin{array}{l}{\zeta }_{mn}{{\rm{J}}}_{m}({\zeta }_{mn}\tilde{a}){{\rm{J}}}_{m-1}({\zeta }_{mn{\prime} }\tilde{a})\hfill \\ -{\zeta }_{mn{\prime} }{{\rm{J}}}_{m}({\zeta }_{mn{\prime} }\tilde{a}){{\rm{J}}}_{m-1}({\zeta }_{mn}\tilde{a})\end{array}\right\}\quad {\text{for}}\ n\, \ne \, n',\hfill \\ \frac{\tilde{a}}{2{\zeta }_{mn}}\left\{\begin{array}{l}\tilde{a}{\zeta }_{mn}{{\rm{J}}}_{m}^{2}({\zeta }_{mn}\tilde{a})+\tilde{a}{\zeta }_{mn}{{\rm{J}}}_{m-1}^{2}({\zeta }_{mn}\tilde{a}) \\ -2(m-1){{\rm{J}}}_{m}({\zeta }_{mn}\tilde{a}){{\rm{J}}}_{m-1}({\zeta }_{mn}\tilde{a})\end{array}\right\}\quad {\text{for}}\ n=n',\end{array}\right. \\ {Y}_{nn{\prime}} 	={\delta }_{nn{\prime} }\frac{1}{2}{{\rm{J}}}_{m-1}^{2}({\zeta }_{mn}),$$

where $$\tilde{a}\equiv a/R$$ and $$\tilde{r}\equiv r/R$$.

### Topological properties at large momentum

In our MP system with a sign-changing magnetic field, the bulk-boundary correspondence guarantees the existence of two kind of gapless topological kink modes, termed as the even and odd modes, respectively. The even modes show gapless feature at the momentum zero, whereas the odd modes show gapless feature at the momentum infinity. They both connect to a zero-frequency bulk band (see ref. ^5^ for detailed analysis).

**Fig. 7:**
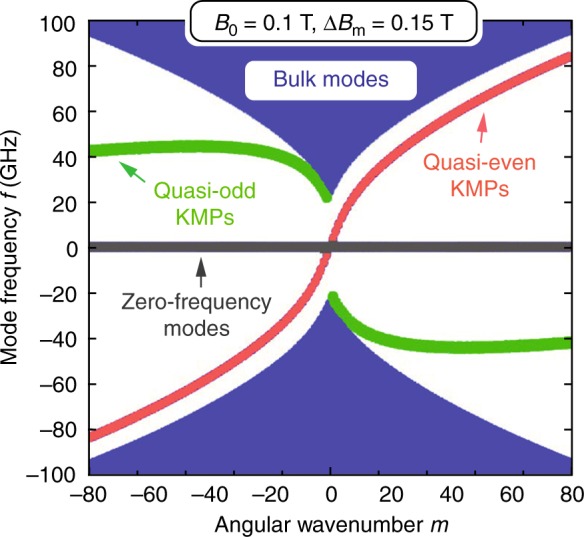
Dispersion of kink magnetoplasmons (KMPs) with large angular wavenumber. The dispersion for ${B}_{0}=0.1$ T, $\Delta B_{\mathrm{m}}=0.15$ T, $\bar{B}_{\mathrm{m}}=0$ T, and ${n}_{0}=1\times 1{0}^{11}\ {{\rm{cm}}}^{-2}$ with large range of angular wavenumber $|m|$ up to 80. The quasi-odd modes first go up and then go down

In the case when the magnetic field is imbalanced in the two domains as in Fig. 2c, by a quick glance, it is puzzling that the odd modes seem not to go down towards the zero-frequency band. This triggers a question on whether such modes are topological or not. But a more refined calculation in a much larger (angular) momentum range shows that they eventually do bend downward and make themselves gapless at the (angular) momentum infinity. This restores all the necessary topological requirements. Fig. 7 displays this behavior.

#### Impact of loss and non-hermiticity

The finiteness of the electronic damping rate $\gamma$ unavoidably introduces a finite linewidth and lifetime to all MP modes (bulk, edge, or kink). The topological protection of KMPs assumes hermicity; additionally, it cannot suppress loss due to non-Hermitian perturbations, such as intrinsic electronic decay (due to imperfections, electron–electron, and electron–phonon scattering).

A natural question is how does intrinsic loss, or more generally non-hermiticity of the Hamiltonian, influence the topological structure^45,46^. It has been shown that the definition of Chern number needs to be generalized^45^. Consider a 2D Dirac-cone structure with a two-fold degeneracy point (closely related to our MP problem)^5,17^. After breaking the degeneracy by opening a gap and in the meanwhile generalizing the parameter space into a complex plane, the eigenenergies and eigenstates reside on a two-Riemann-sheet manifold. The two sheets are connected by two square-root branch cuts ended at two exceptional points (EPs)^47^. The eigenstates, following the general formalism of non-Hermitian Hamiltonian, can be divided into the left-eigenstates $|{\mathcal{A}}{> }^{\text{L}}$ and right-eigenstates $|{\mathcal{A}}{> }^{\text{R}}$. They all together form four Chern numbers ${C}^{\text{LL}}$, ${C}^{\text{LR}}$, ${C}^{\text{RL}}$, ${C}^{\text{RR}}$, which can be proved to be all equal^45^. As long as the gap remains open in the complex-energy plane, the topological edge modes persist.

A related, more practical question is how much loss is required to effectively close the bandgap (filling appreciable density of states into the gap). For our 2DEG samples, the high mobility $\mu \sim 1{0}^{7}\ {{\rm{cm}}}^{2}$ V^−1^ entails a low damping rate $\gamma =e/{m}_{* }\mu \approx 2.6$ GHz. It is the ratio $\gamma /\omega_{\mathrm{c}}$ that controls the loss-induced gap closing. First of all, the governing Eq. (2) must be modified to include the loss rate $\gamma$,

16 $$(\omega +{\rm{i}}\gamma ){\bf{j}}({\bf{r}},\omega )=-{\rm{i}}\frac{{e}^{2}}{{m}_{* }}n({\bf{r}})\nabla \Phi ({\bf{r}},\omega )-{\rm{i}}\omega_{\mathrm{c}}({\bf{r}}){\bf{j}}({\bf{r}},\omega )\times \hat{{\bf{e}}}_{z}.$$

It is sufficient to consider the uniform bulk at long-wavelength limit only. The (non-Hermitian) Hamiltonian equation is

17 $$\begin{array}{*{20}{l}}{\omega} \left(\begin{array}{c}{j}_{\rho }\\ {j}_{x}\\ {j}_{y}\end{array}\right) ={\mathcal{H}}\left(\begin{array}{c}{j}_{\rho }\\ {j}_{x} \\ {j}_{y}\end{array}\right)=\left(\begin{array}{ccc}0&{v}_{\text{p}}{q}_{x}&{v}_{\text{p}}{q}_{y}\\ {v}_{\text{p}}{q}_{x}&-{\rm{i}}{\gamma} &-{\rm{i}}{\omega}_{\mathrm{c}}\\ {v}_{\text{p}}{q}_{y}&+{\rm{i}}{\omega}_{\mathrm{c}}&-{\rm{i}}{\gamma} \end{array}\right)\left(\begin{array}{c}{j}_{\rho }\\ {j}_{x}\\ {j}_{y}\end{array}\right),\end{array}$$

where ${j}_{\rho }\equiv {v}_{\text{p}}\rho$, and ${v}_{\text{p}}$ is an effective plasmon velocity^5^,

18 $${v}_{\text{p}}=\sqrt{\frac{4\pi {e}^{2}{n}_{0}}{{m}_{* }}\frac{d_{\mathrm{{A}}}d_{\mathrm{{B}}}}{\varepsilon_{\mathrm{{A}}}d_{\mathrm{{B}}}+\varepsilon_{\mathrm{{B}}}d_{\mathrm{{B}}}}}.$$

For a lossy system, it is more physical to plot the response function instead of the dispersion relation. The Green operator (response matrix) of this problem is

19 $${\mathcal{G}}({\bf{q}},\omega )={\left[\omega {\mathcal{I}}-{\mathcal{H}}({\bf{q}},\omega )\right]}^{-1},$$

which can be derived analytically. For example, the ${G}_{{j}_{y}{j}_{x}}$ component, which gives the response in ${j}_{y}$ due to a source in ${j}_{x}$, is

20 $${G}_{{j}_{y}{j}_{x}}({\bf{q}},\omega )=-\frac{{v}_{\text{p}}^{2}{q}_{x}{q}_{y}+{\rm{i}}\omega \omega_{\mathrm{c}}}{\omega \left(\right.\omega_{\mathrm{c}}^{2}-{({\omega }^{2}+{\rm{i}}\gamma )}^{2}\left)\right.+{v}_{\text{p}}^{2}({q}_{x}^{2}+{q}_{y}^{2})(\omega +{\rm{i}}\gamma )}.$$

**Fig. 8:**
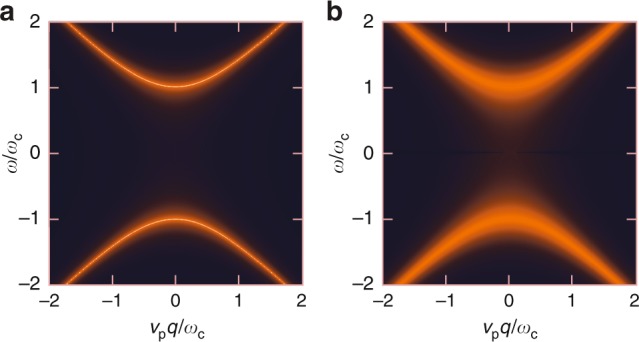
Impact of loss on magnetoplasmonic dispersion. Color plot of response function ${G}_{{j}_{y}{j}_{x}}({\bf{q}}=q\hat{{\bf{e}}}_{\mathrm{x}},\omega )$ for different loss factors. **a** $\gamma /\omega_{\mathrm{c}}=5\times 1{0}^{-5}$, negligible loss, representing the ideal case. **b** $\gamma /\omega_{\mathrm{c}}=5\times 1{0}^{-2}$, finite but small loss, representative of our samples

As noted previously, in our setup $\omega_{\mathrm{c}}\approx 50$ GHz and $\gamma \approx 2.6$ GHz, such that $\gamma \sim 5\times 1{0}^{-2}\omega_{\mathrm{c}}$, rendering the time-reversal-breaking scale ~$20$ times greater than the non-Hermitian scale. In Fig. 8, we plot the response function in the $({\bf{q}}=q\hat{{\bf{e}}}_{\mathrm{x}},\omega )$ plane. For comparison, we choose $\gamma /\omega_{\mathrm{c}}=5\times 1{0}^{-5}$ for nearly vanishing loss and $\gamma /\omega_{\mathrm{c}}=5\times 1{0}^{-2}$ for our samples. The two plots use the same color scale. The latter has slightly blurred band edges, but the gap is still well-defined and open.

### Experiments with different samples and magnets

To further verify the properties of KMPs, we have performed experiments with different 2DEG samples and different NdFeB magnets. For instance, we use a sample of much higher electron concentration ${n}_{0}=2.5\times 1{0}^{11}\ {{\rm{cm}}}^{-2}$ (at zero gate voltage) and higher electron mobility $\mu =1.46\times 1{0}^{7}\ {{\rm{cm}}}^{2}$ V^−1^ s^−1^ which also produce well-defined KMP resonances. The increased electron density causes a large overall blueshift of KMP resonance frequencies relative to Fig. 3; as a result, only the $m=1$ quasi-even mode remains within our 1–9 GHz reliable measurement range. Additionally, the increased mobility (and concomitantly reduced loss) produces significantly sharper resonances.

**Fig. 9:**
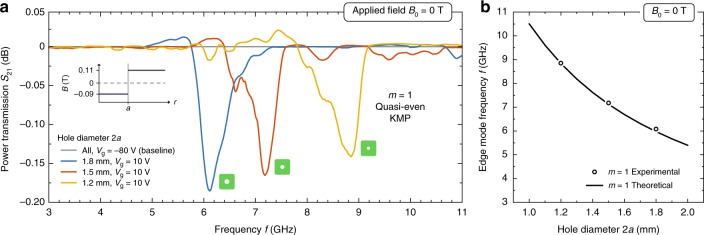
Kink magnetoplasmons (KMPs) for distinct magnetic hole diameters. **a** Measured power transmission ${S}_{21}$ (normalized to indicated baselines) as a function of frequency for the fixed applied magnetic field ${B}_{0}=\text{0 T}$ and gate voltage $V_{\mathrm{g}}=10$ for each signal and $-80$ V for each baseline. **b** Comparison between experimental observations and theoretical calculations, ${n}_{0}=\text{2.64}\times {\text{10}}^{\text{11}}\ {{\rm{cm}}}^{-\text{2}}$ (at 10 V gate voltage)

We place on top of this sample differently customized magnets of varying hole diameters $2a=1.2$, 1.5, and 1.8 mm. The spacer and substrate thicknesses are $d_{\mathrm{A}}=d_{\mathrm{B}}=200\,\mu$m. As expected, with increasing hole diameter, i.e., increasing length of the domain boundary, the resonance frequency decreases. The location of the absorption dips are consistent with the theoretical calculation. These results are summarized in Fig. 9.

**Fig. 10:**
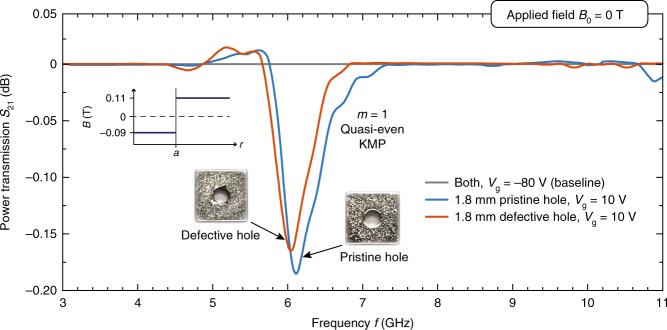
Kink magnetoplasmons (KMPs) in magnetic domain with defects. Measured power transmission ${S}_{21}$ (normalized to indicated baselines) as a function of frequency for topological kink magnetoplasmons (KMPs) along defective and pristine magnetic domain boundaries

We then verify the existence of resonances in the presence of defects along the edge. This will be a strong indication of suppressed back scattering and topological protection. By knocking off a few chips from the upper hole edge of the magnet, we make a small local perturbation to the field profile projected onto the 2DEG underneath. The measured results are given in Fig. 10. The defect only produces a minor frequency shift and negligible transmission reduction. Evidently, the overall absorption structure is not significantly perturbed relative to the pristine configuration.

## Supplementary information

Supplementary Information

## Data Availability

All relevant data is available from the authors upon request.
