# Supplementary material for: Topological kink plasmons on magnetic-domain boundaries
Source: Nat Commun. 2019 Oct 8;10:4565. doi: 10.1038/s41467-019-12092-x (PMC6783483; doi:10.1038/s41467-019-12092-x)
Supplement: Supplementary file 1 — Supplementary Information [file 41467_2019_12092_MOESM1_ESM.pdf]

Supplementary Information

Topological kink plasmons on magnetic-domain boundaries

Jin *et al.*

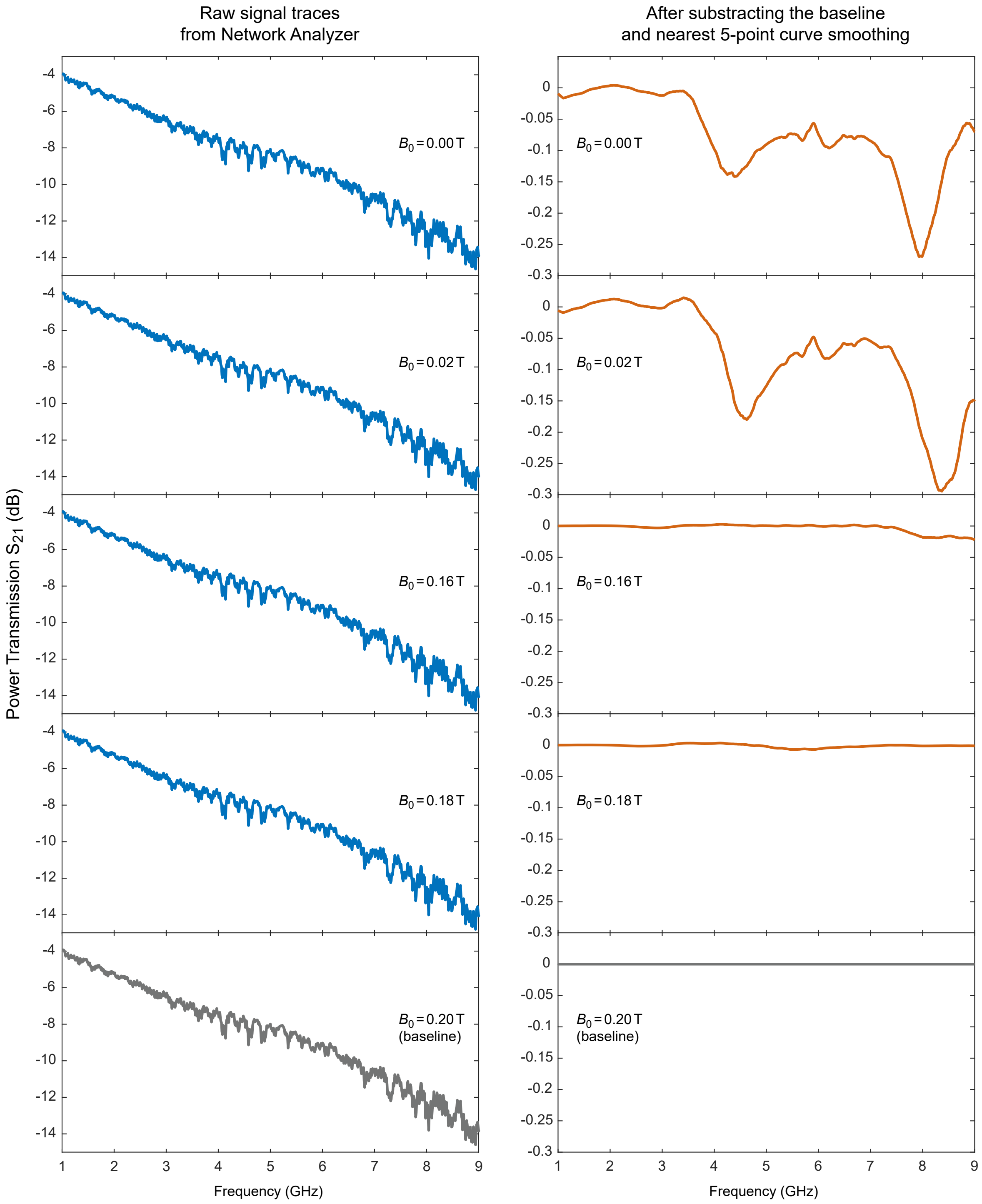

Supplementary Figure 1. Comparison of raw microwave signal traces from network analyzer without taking a proper baseline and processed data after subtracting the baseline and nearest 5-point curve smoothing.
